# miR‐33 in cardiometabolic diseases: lessons learned from novel animal models and approaches

**DOI:** 10.15252/emmm.202012606

**Published:** 2021-05-03

**Authors:** Nathan L Price, Leigh Goedeke, Yajaira Suárez, Carlos Fernández‐Hernando

**Affiliations:** ^1^ Vascular Biology and Therapeutics Program Yale University School of Medicine New Haven CT USA; ^2^ Department of Comparative Medicine Integrative Cell Signaling and Neurobiology of Metabolism Program Yale University School of Medicine New Haven CT USA; ^3^ Department of Internal Medicine Yale University School of Medicine New Haven CT USA; ^4^ Department of Pathology Yale University School of Medicine New Haven CT USA

**Keywords:** atherosclerosis, metabolism, miR‐33, miRNA, Cardiovascular System, Chromatin, Epigenetics, Genomics & Functional Genomics, Metabolism

## Abstract

miRNAs have emerged as critical regulators of nearly all biologic processes and important therapeutic targets for numerous diseases. However, despite the tremendous progress that has been made in this field, many misconceptions remain among much of the broader scientific community about the manner in which miRNAs function. In this review, we focus on miR‐33, one of the most extensively studied miRNAs, as an example, to highlight many of the advances that have been made in the miRNA field and the hurdles that must be cleared to promote the development of miRNA‐based therapies. We discuss how the generation of novel animal models and newly developed experimental techniques helped to elucidate the specialized roles of miR‐33 within different tissues and begin to define the specific mechanisms by which miR‐33 contributes to cardiometabolic diseases including obesity and atherosclerosis. This review will summarize what is known about miR‐33 and highlight common obstacles in the miRNA field and then describe recent advances and approaches that have allowed researchers to provide a more complete picture of the specific functions of this miRNA.

## Graphical abstract

miRNAs have emerged as critical regulators of biologic processes and important therapeutic targets for numerous diseases. This review focusses on miR‐33 as an example, and describes the obstacles, recent advances and new approaches in the miRNA field.

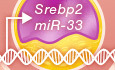

**Glossary**
Atherosclerosis: Vascular disease characterized by the progressive accumulation of lipids and inflammatory cells in the subendothelial space of large arteries.
Binding site mutant (BSM): Targeted modifications of miRNA binding sites to selectively disrupt specific miRNA/target interactions.
Cholesterol Efflux: Process by which peripheral tissues transfer intracellular cholesterol onto extracellular acceptors like high‐density lipoprotein cholesterol (HDL).
High‐density lipoproteins (HDL): Lipoprotein particles that promote the cellular cholesterol efflux from cells and deliver cholesterol to the liver and steroidogenic tissues via selective lipid uptake.
microRNA (miRNA): Small non‐coding RNA that negatively regulates protein expression by promoting mRNA degradation or impairing protein translation.
pH low insertion peptides (pHLIP): Constructs designed to deliver small amounts of nuclear material only to acidic microenvironments.
Reverse Cholesterol Transport (RCT): Process by which cholesterol is removed from peripheral tissues and transported to the liver for removal or redistribution.
RNA‐induced Silencing Complex (RISC): Multiprotein complex that facilitates targeted inhibitory activity of small regulatory RNAs like miRNAs and siRNAs.

## Introduction

Since the structure of DNA was uncovered in 1953, the central dogma of biology—that cellular functions are carried out by transcription of DNA into RNA, which is then translated into the proteins that carry out physiologic functions—has remained largely unchanged. Under this theory, regions of the DNA that do not code for proteins are largely non‐functional. However, with the discovery of miRNAs in 1993 (Lee *et al,* 1993; Wightman *et al,* 1993), our understanding of the mechanisms of biologic regulation has evolved dramatically. Some of the early work on miRNAs suggested that these novel regulatory molecules may function primarily as “fine tuners” of biologic functions and many researchers, as well as members of the general public still hold this belief. However, extensive work has been done that clearly demonstrates that disruption of miRNA processing or alterations in individual miRNAs can have a profound effect on many different cellular functions (Bernstein *et al,* 2003; Harfe *et al,* 2005; Rodriguez *et al,* 2007; Zhao *et al,* 2007). Indeed, miRNAs have emerged as major regulators of nearly all biologic functions, as well as the development of numerous different diseases. miRNAs are also known to be dysregulated in different disease states and are able to regulate cellular functions related to disease progression. As such, they have emerged as promising targets for the development of novel therapeutic approaches.

miRNAs are encoded both in non‐coding regions of the genome and within introns of protein‐coding genes. Primary miRNA sequences are transcribed by the RNA polymerase II enzyme, after which they undergo sequential cleavage by the Drosha and Dicer enzymes to yield small single‐stranded RNA molecules (~22 nt) that can be incorporated into the RNA‐induced silencing complex (RISC). Once incorporated into the RISC, miRNAs facilitate binding of a distinct subset of mRNA targets by targeting specific sequences, generally within the 3’ untranslated regions (3’UTR), leading to either mRNA degradation or impaired translation (Ambros, 2004; Bartel, 2009). One of the ways in which miRNAs are able to exert such robust effects is by inhibition of multiple different mRNAs within the same or related pathways. While the ability of miRNAs to target many different mRNAs contributes to their ability to exert both very pronounced and extremely nuanced effects in different situations, this promiscuity has also raised a number of important issues in terms of both research on and clinical applications of miRNAs. These difficulties are further enhanced by the fact that the impact and target preferences of miRNAs can vary dramatically between different tissues and cell types. Many factors contribute to this, including the expression of the miRNA of interest, the relative levels of various miRNA targets, and the presence of a number of different molecules (long non‐coding RNAs (lncRNAs), circular RNAs, RNA‐binding proteins, mRNAs) that can act as miRNA sponges.

The combination of these factors has made determining the specific mechanisms by which miRNAs exert their effects very challenging. For many years, researchers have relied on a limited toolset with which to identify and validate miRNA targets. A number of different target prediction algorithms have been developed that can be used identify which mRNAs are likely targets for a specific miRNA, and this targeting can then be validated using 3’UTR luciferase assays. However, assessing the likelihood that a miRNA target is involved in mediating a specific function has largely relied on associations between mRNA/protein levels and alterations in miRNA expression. In recent years, a great deal of progress has been made in the development and implementation of new tools and techniques that allow researchers to directly assess the binding of mRNAs to the RISC and specific miRNAs, determine the relative impact of individual miRNA/target interactions, and evaluate the specific role of miRNAs in different tissues and/or cell types. These new strategies will help address many of the main obstacles facing the development of miRNA‐based therapies by improving our ability to determine the specific mechanisms by which miRNAs exert their effects and assess potential detrimental outcomes of miRNA‐based therapies.

miR‐33 is one of the most well‐studied miRNAs and is an excellent example of the potential of miRNA‐based therapeutics to treat a variety of different human diseases, the possible hazards that may be involved in miRNA‐based therapeutic approaches, and the techniques that are being developed to try to better understand and minimize these risks. In this review, we will summarize the work that was done to characterize the role of miR‐33 in metabolism, atherosclerosis, and other conditions. We will then describe in detail the recent advances that have been made in understanding the specific functions of miR‐33 with a focus upon the utilization of new tools for the study of miRNA biology, including tissue‐specific mouse models, new approaches to disrupt specific miRNA/target interactions, and targeted miRNA therapies.

## Regulation of lipid metabolism by the miR‐33/SREBP/LXR axis

### Reciprocal regulation of cholesterol and fatty acid metabolism

Proper control of lipid homeostasis is crucial for the maintenance of human physiology and health (Rottiers & Naar, 2012). Accordingly, intricate regulatory networks have evolved to monitor and respond to metabolic and hormonal cues. Work over the past decades has suggested that much of the orchestration of these responses occurs at the level of gene regulation in the cell nucleus. In particular, two transcription factor families—the sterol response element binding proteins (SREBPs) and liver X receptors (LXRs)—help maintain metabolic homeostasis by governing complex gene expression programs that control the production, uptake, and output of lipids (DeBose‐Boyd & Ye, 2018; Wang & Tontonoz, 2018).

The SREBP family of basic helix–loop–helix leucine zipper transcription factors directly activate the expression of more than 30 genes involved in cholesterol, fatty acid, triglyceride, and phospholipid metabolism in the liver (DeBose‐Boyd & Ye, 2018). The mammalian genome contains three SREBP isoforms, designated SREBP1a and SREBP1c, encoded by the *SREBF1* gene, and SREBP2, encoded by *SREBF2*. The SREBPs differ in their tissue‐specific expression, their target gene selectivity, and the relative potency of their transactivation domains (Brown & Goldstein, 1997). Although there is some functional overlap between different isoforms, sterol‐responsive SREBP2 mostly activates genes involved in cholesterol biosynthesis and uptake (e.g., *HMGCR* and *LDLR*), whereas insulin‐responsive SREBP1c is more selective for genes involved in fatty acid metabolism and lipogenesis (e.g., *FASN* and *ACC*) (Horton *et al,* 2002).

The LXRs (LXRα and LXRβ) are nuclear hormone receptors that form active heterodimers with retinoid X receptors (RXRs) and are activated by a variety of sterols, including oxysterol intermediates that form during cholesterol biosynthesis (Peet *et al,* 1998). In response to elevated sterols, LXRs function to induce the expression of genes critical to cholesterol efflux (*ABCA1, ABCG1*), cholesterol conversion to bile acids (*CYP7A1*), and cholesterol secretion into bile (*ABCG5/G8*) (Wang & Tontonoz, 2018). In this regard, abrogation of LXR expression in mouse models results in cholesterol accumulation and accelerated atherosclerosis, whereas pharmacological activation of LXRs with synthetic agonists confers protection (Claudel *et al,* 2001; Joseph *et al,* 2002; Bradley *et al,* 2007). LXRs also enhance the expression of SREBP1c and lipogenesis in mice (Repa *et al,* 2000; Schultz *et al,* 2000), thereby mediating crosstalk between these two transcription factor families to ensure continued supply of fatty acids for esterification of free cholesterol. Indeed, it was recently shown that mice lacking *Srebp2* in hepatocytes and hypomorphic *Srebp2* mice have reduced SREBP1c and lipogenic gene expression (Vergnes *et al,* 2016; Naar, 2018), suggesting that flux through the cholesterol and fatty acid pathway in the liver are tightly linked.

### miR‐33/SREBP/LXR axis

**Figure 1:**
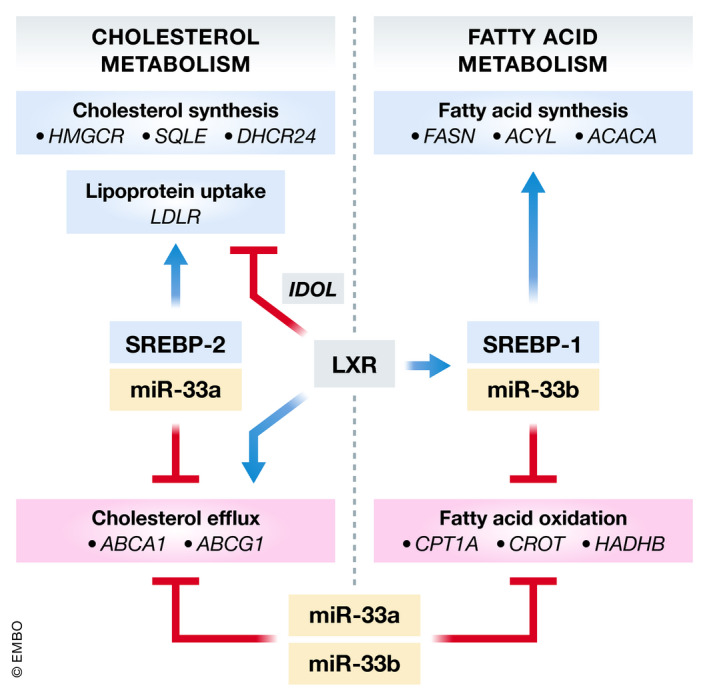
Regulation of cholesterol and fatty acid metabolism by the SREBP and LXR transcription factors and miR‐33 family members Schematic diagram depicting the synergistic relationship between *miR‐33a/b*, their host genes (*SREBF2* and *SREBF1),* and *LXR*. Feed‐forward regulation of SREBP2 and SREBP1 by low sterol levels or LXR ligands/insulin results in co‐transcription of miR‐33a and miR‐33b. While SREBP2 and SREBP1 promote the transcription of genes involved in cholesterol synthesis/uptake and fatty acid synthesis, respectively, miR‐33a/b inhibit the expression of genes involved in cholesterol transport and fatty acid metabolism, resulting in reduced fatty acid oxidation and decreased cholesterol efflux.

In 2004, Rodriguez et al (2004) identified *miR‐33*, an intronic miRNA embedded within intron 16 of the human *SREBF* gene. Several years later, the functional consequence of this location was revealed, adding to the complexity of crosstalk between the SREBP and LXR transcription families (Najafi‐Shoushtari *et al,* 2010; Rayner *et al,* 2010). Feed‐forward regulation of SREBP leads to the expression of two closely related miRNAs (*miR‐33a* and *miR‐33b*) that are encoded within introns 16 and 17 of the human *SREBF2* and *SREBF1* genes, respectively (Gerin *et al,* 2010; Horie *et al,* 2010; Marquart *et al,* 2010; Najafi‐Shoushtari *et al,* 2010; Rayner *et al,* 2010). Despite a two‐nucleotide variation in the mature forms of miR‐33a and miR‐33b, multiple studies have revealed that these miRNAs (and their passenger strands, miR‐33a*/b*) are co‐expressed with SREBPs in many different cell types and tissues and work together with their host genes to control lipid homeostasis (Gerin *et al,* 2010; Horie *et al,* 2010; Marquart *et al,* 2010; Najafi‐Shoushtari *et al,* 2010; Rayner *et al,* 2010; Davalos *et al,* 2011; Goedeke *et al,* 2013; Shao *et al,* 2014; Shao *et al,* 2019; Zhu *et al,* 2020). For example, one of the main targets of miR‐33 is ABCA1, an ATP‐binding cassette transporter that mediates cholesterol efflux to lipid‐poor ApoA1 and ApoE (Oram, 2000). During sterol‐depleted conditions, when SREBP2 is activated to increase cholesterol biosynthesis and uptake, miR‐33 prevents the efflux of newly synthesized cholesterol from cells by targeting ABCA1. Conversely, when cellular cholesterol levels are high, SREBP2 processing is inhibited, thereby decreasing cholesterol biosynthesis and uptake and reducing the expression of miR‐33 (Gerin *et al,* 2010; Horie *et al,* 2010; Marquart *et al,* 2010; Najafi‐Shoushtari *et al,* 2010; Rayner *et al,* 2010). Interestingly, LXRs are also activated in this state and work with the SREBP2/miR‐33 axis to increase cholesterol efflux by activating the expression of ABCA1 (Fig 1).

In addition to the coordinated regulation of cholesterol levels by miR‐33a/b and SREBP2, miR‐33 also controls intracellular fatty acid and lipid levels in concert with its *SREBF1* host gene by targeting genes involved in fatty acid oxidation (*CPT1a*, *CROT*, *HADHB, AMPK*) (Gerin *et al,* 2010; Davalos *et al,* 2011; Shao *et al,* 2019) (Fig 1). Interestingly, while miR‐33a is evolutionarily conserved across multiple species, rodents lack *miR‐33b* in the corresponding intron of *Srebp1*. The significance of this conservation is important, as it could help explain why individuals with insulin resistance have high VLDL‐triglyceride levels and low HDL‐cholesterol levels (Brown *et al,* 2010). Indeed, several studies have verified that hepatic miR‐33b levels are increased in human cell lines and non‐human primates in response to nutrient excess (Davalos *et al,* 2011; Goedeke *et al,* 2013; Ramirez *et al,* 2013) and show that anti‐sense inhibitors of miR‐33a/b are effective at increasing HDL levels (Rayner *et al,* 2011a; Rottiers *et al,* 2013; Cai *et al,* 2019) and lowering VLDL triglycerides in non‐human primates (Rayner *et al,* 2011a). The synergistic functions of miR‐33a and miR‐33b with their host genes help to further amplify the critical physiologic responses of these key regulators of lipid metabolism, but work over the past decade has also demonstrated that dysregulation of these miRNAs can contribute to the development of many different diseases.

## Relevance of miR‐33 in cardiometabolic disorders and other diseases

### miR‐33 and atherosclerosis

**Figure 2:**
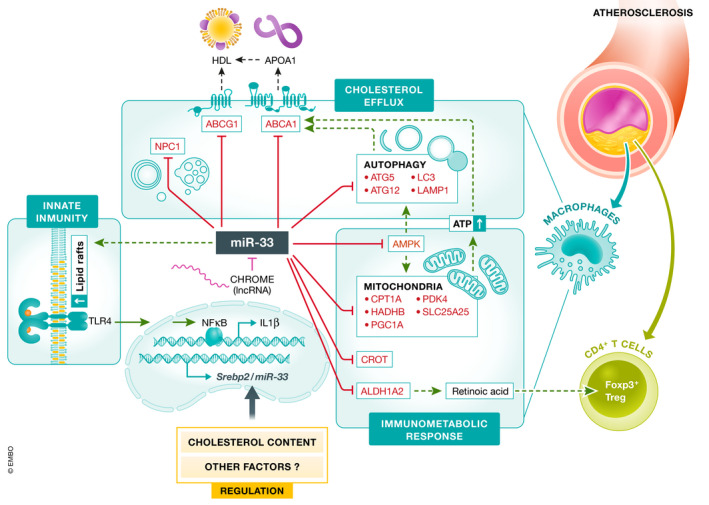
miR‐33‐mediated regulation of macrophage immunometabolism in atherosclerotic lesions Schematic overview showing the metabolic pathways regulated by miR‐33 in macrophages. Macrophage miR‐33 levels are regulated by intracellular cholesterol content and other factors, including the lncRNA, CHROME. miR‐33 controls the expression of genes associated with cellular cholesterol efflux (ABCA1, ABCG1, NPC1), fatty acid oxidation (CPT1A, HADHB, CROT), mitochondrial biogenesis (AMPK, PGC1A, PDK4, SLC25A25), and autophagy (ATG5, ATG12, LC3, LAMP1), which modulate macrophage fate and the innate immune response. Additionally, miR‐33 expression regulates retinoic acid production (by targeting ALDH1A2) which controls T‐cell responses, promoting an anti‐inflammatory and resolving microenvironment in atherosclerotic plaques.

**Figure 3:**
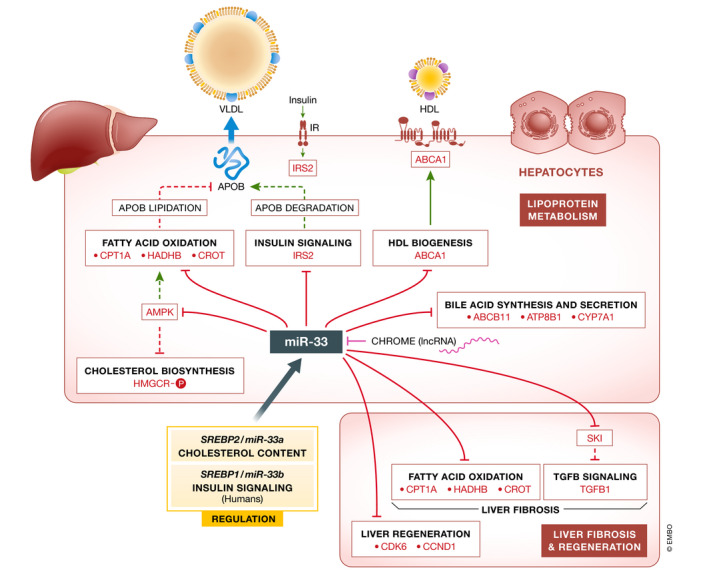
miR‐33 regulates hepatic lipoprotein metabolism, fibrosis, and regeneration Schematic overview showing the metabolic pathways regulated by miR‐33 in hepatocytes. Hepatic miR‐33 levels are regulated by cellular cholesterol content, insulin, and other factors, including lncRNA CHROME. miR‐33 controls numerous steps of the reverse cholesterol transport pathway, such as HDL biogenesis (ABCA1) and bile acid synthesis (ABCB11, ATP8B1, CYP7A1). miR‐33 also regulates the expression of numerous genes associated with fatty acid oxidation (CPT1A, HADHB, CROT), which might influence ApoB lipidation and VLDL secretion, and glucose metabolism (IRS2, PCK1, G6PC). Suppression of hepatic miR‐33 attenuates obesity‐driven liver fibrosis and promotes hepatic regeneration by enhancing fatty acid oxidation and SKI levels and increasing the expression of cell cycle regulators (CDK6 and CCND1), respectively.

The strong inverse correlation between low circulating HDL‐C levels and cardiovascular disease (CVD) risk in human epidemiological studies has led to a great deal of work exploring the potential of inhibiting miR‐33 in prophylactic and therapeutic murine models of atherosclerosis. Inhibition or deletion of miR‐33 in Western diet (WD)‐fed *Ldlr^‐/‐^* or *Apoe* ^‐/‐^ mice was found to have beneficial outcomes, demonstrating that loss of miR‐33 can reduce atherosclerotic plaque size and promote atherosclerosis regression (Rayner *et al,* 2011b; Horie *et al,* 2012; Rotllan *et al,* 2013; Distel *et al,* 2014; Karunakaran *et al,* 2015; Ouimet *et al,* 2015; Price *et al,* 2017). Most of these effects have been attributed to miR‐33’s ability to increase circulating levels of HDL and/or promote macrophage cholesterol efflux. However, recent studies have shown that genetic or chemical inhibition of miR‐33 in WD‐fed *Ldlr* ^‐/‐^ mice was atheroprotective even in the absence of changes in circulating HDL (Rotllan *et al,* 2013; Price *et al,* 2017), indicating that miR‐33 has anti‐atherogenic properties that are distinct from its HDL‐raising capabilities. Indeed, several lines of evidence indicate that miR‐33 is involved in regulating numerous processes related to CVD, including cholesterol metabolism, autophagy, macrophage polarization, fatty acid metabolism, and insulin signaling/glucose homeostasis (Figs 2 and 3).

#### Regulation of reverse cholesterol transport by miR‐33

Given the essential role of ABCA1 in regulating HDL biogenesis reverse and cholesterol transport (RCT), the process by which mammalian cells orchestrate the removal of excess cholesterol from peripheral tissues to the liver (Tall *et al,* 2008), numerous studies have investigated the ability of miR‐33 to regulate these processes *in vitro* and *in vivo*. Initially, it was shown that miR‐33 can reduce cholesterol efflux to ApoA1 and HDL by modulating the expression of ABCA1 and ABCG1 (rodents only) in macrophages. Importantly, inhibition of endogenous miR‐33 results in increased expression of the ABCA1 protein and a concomitant increase in cholesterol efflux, indicating a physiologic role for this miRNA in regulating ABCA1 expression (Gerin *et al,* 2010; Horie *et al,* 2010; Marquart *et al,* 2010; Najafi‐Shoushtari *et al,* 2010; Rayner *et al,* 2010). Further work has gone on to show that miR‐33a/b control HDL‐C biogenesis by targeting ABCA1 in the liver and are involved in the last stage of RCT by regulating factors involved in the synthesis (CYP7A1) and secretion (ATP8B1 and ABCB11) of bile acids (Rayner *et al,* 2011b; Allen *et al,* 2012; Li *et al,* 2013; Tarling *et al,* 2015). Together these studies demonstrate that miR‐33 is an important regulator of multiple steps in the RCT pathway. Indeed, miR‐33b knock‐in mice (Horie *et al,* 2014) were shown to have lower HDL‐C levels compared to wild‐type controls, while antagonism of miR‐33 results in increased hepatic ABCA1 expression and elevated HDL‐C levels in mice and non‐human primates (Najafi‐Shoushtari *et al,* 2010; Rayner *et al,* 2010; Rayner *et al,* 2011a; Rayner *et al,* 2011b; Rottiers *et al,* 2013; Karunakaran *et al,* 2015; Cai *et al,* 2019).

Intriguingly, miR‐33 also regulates other functions that indirectly impact cholesterol efflux. miR‐33 regulates intracellular cholesterol trafficking by targeting a number of different factors, including NPC1, OSBPL6, and NSF (Rayner *et al,* 2010; Marquart *et al,* 2013; Ouimet *et al,* 2016a), which can impact the availability of cholesterol for cellular efflux. While it remains to be determined whether miR‐33‐mediated silencing of OSBPL6 affects cholesterol efflux to HDL‐C levels *in vivo*, OSBPL6 mRNA levels positively correlate with ApoA1 and HDL‐C levels in humans and OSBPL6 levels are decreased in the carotid arteries of patients with atherosclerosis (Ouimet *et al,* 2016a). Similarly, miR‐33 may affect RCT by controlling autophagy, a process that is critical for the mobilization of free cholesterol for macrophage efflux (Ouimet *et al,* 2019). In 2016 and 2017, Oiumet *et al* demonstrated that miR‐33 reduces lipid droplet catabolism in macrophage foam cells by repressing key autophagy effectors (*Atg5, Atg12, Map1* *lc3b, Prkaa1, and Lamp1*), as well as their transcriptional regulators (*Foxo3* and *Tfeb*) (Ouimet *et al,* 2016b; Ouimet *et al,* 2017). Notably, the authors show that the ability of anti‐miR‐33 to enhance cholesterol efflux is lost in macrophages chemically or genetically deficient in autophagy, demonstrating a role for this pathway in miR‐33’s atheroprotective effects (Ouimet *et al,* 2017). Additionally, miR‐33 has been demonstrated to target a number of factors involved in mitochondrial biogenesis, mitochondrial respiration, and ATP production, including AMPK, PGC1α, PDK4, and SLC25A25 (Karunakaran *et al,* 2015). As the function of cholesterol transporters, such as ABCA1 and ABCG1, requires ATP, impairment in mitochondrial function has been shown to limit the ability of miR‐33 inhibitors to improve cholesterol efflux capacity. While these findings demonstrate that the disruption of pathways related to autophagy and mitochondrial function can limit the ability of miR‐33 deficiency/inhibition to increase cholesterol efflux, these studies do not directly test whether the repression of these factors is required for the adverse effects of miR‐33 on RCT and CVD. Together these findings demonstrate that in addition to the direct effects of miR‐33 on RCT, this miRNA can also indirectly impede this process by targeting factors involved in ATP production and cholesterol availability (Fig 2).

#### Regulation of other cellular functions related to atherosclerosis

In addition to regulation of RCT, miR‐33 has been shown to impact a number of other important cellular functions of the cells that make up the arterial wall and participate in atherosclerosis, including endothelial cells (ECs), vascular smooth muscle cells (VSMCs), and/or macrophages. As discussed, miR‐33 is an important regulator of fatty acid metabolism and cellular bioenergetics and can also play an important role in regulation of insulin signaling and glucose metabolism (Gerin *et al,* 2010; Davalos *et al,* 2011; Ramirez *et al,* 2013), all of which can have an important impact on cellular functions. For example, Oiumet *et al* showed that miR‐33 inhibitors are able to transduce aortic root macrophages in *Ldlr^‐/‐^* mice and promote their polarization toward an anti‐inflammatory M2 phenotype (Ouimet *et al,* 2015) through targeting of AMPK. miR‐33 has also been shown to repress SIRT6, a NAD+‐dependent protein deacetylase that has previously been implicated in modulating inflammation and macrophage phenotypes (Davalos *et al,* 2011). Consistent with these findings, in mouse models of atherosclerosis and abdominal aortic aneurysms genetic and chemical inhibition of miR‐33 reduces macrophage inflammation (Rayner *et al,* 2011b; Rotllan *et al,* 2013; Distel *et al,* 2014; Ouimet *et al,* 2015; Price *et al,* 2017) and promotes the expression of anti‐inflammatory M2 makers while reducing expression of pro‐inflammatory M1 markers (Rayner *et al,* 2011b; Distel *et al,* 2014). These effects appear to be context‐dependent, as other studies have shown that miR‐33 can play an anti‐inflammatory role by targeting RIP140, a coactivator for nuclear factor kappa B (NF‐ΚB) (Ho *et al,* 2011; Goedeke *et al,* 2013), and inhibition of miR‐33 was also shown to increase M1 macrophage polarization and prevent age‐related macular degeneration by increasing levels of ABCA1 (Sene *et al,* 2013). Macrophage *Aldh1a2*, a gene involved in retinoic acid metabolism, was also found to be depressed with anti‐miR‐33 treatment, resulting in the induction of regulatory T cells and atheroprotection (Ouimet *et al,* 2015).

The ability of miR‐33 to regulate a number of factors that are both directly and indirectly involved in the process of RCT, as well as numerous other processes that are related to the development of atherosclerosis, is an excellent example of the ability of miRNAs to target many different related factors to produce effects that are more robust than those that could be achieved through the regulation of a single factor or pathway. However, this promiscuous nature of miRNAs can also lead to many other unrelated and in some cases adverse outcomes depending upon the tissue and context in which the miRNA is functioning.

### miR‐33 in obesity and diabetes

#### miR‐33 and insulin signaling/glucose homeostasis

Progress in preventing CVD has been stalled by the growing epidemic of obesity, insulin resistance, and type 2 diabetes (Bornfeldt & Tab as, 2011), which increase the relative risk of developing atherosclerotic vascular disease and its complications fourfold compared to non‐diabetic individuals (Haffner *et al,* 1998; Beckman *et al,* 2002; Gore *et al,* 2015; Hayward *et al,* 2015). In addition to controlling fatty acid metabolism, miR‐33 also regulates insulin signaling and glucose metabolism (Gerin *et al,* 2010; Davalos *et al,* 2011; Ramirez *et al,* 2013). Specifically, multiple groups have demonstrated that miR‐33a/b reduce endogenous IRS‐2 protein levels, thereby inhibiting activation of downstream insulin signaling pathways, including PI3K/AKT (Davalos *et al,* 2011; Rayner *et al,* 2011a; Tang *et al,* 2017). Consistent with this, antagonism of miR‐33 in human liver cell lines and non‐human primates increases hepatic expression of IRS‐2, pAKT/AKT signaling, and glucose uptake (Davalos *et al,* 2011; Rayner *et al,* 2011a). While functionally, this may be predicted to lower plasma glucose levels, no differences were observed in non‐human primates treated with inhibitors of miR‐33a/b (Rottiers *et al,* 2013). Given that Ramirez *et al* demonstrated that miR‐33b also regulates glucose homeostasis through inhibition of PKC1 and G6PC (Ramirez *et al,* 2013), it is possible that anti‐miR‐33‐mediated increases in hepatic gluconeogenesis could mask these effects (Fig 3). Glucose‐stimulated insulin secretion during a glucose tolerance test tended to be increased by anti‐miR‐33 inhibitors in non‐human primates (Rottiers *et al,* 2013), consistent with a prior study suggesting that miR‐33a can regulate insulin secretion in pancreatic islets by targeting ABCA1 expression (Wijesekara *et al,* 2012). Collectively, these studies point toward a role for miR‐33 in regulating a number of genes involved in glucose homeostasis and raise caution about the use of global miR‐33 inhibitors to treat metabolic syndrome.

#### Obesity and metabolic dysfunction of whole‐body miR‐33 KO mice

While initial studies demonstrated the strong potential for miR‐33 inhibitors to serve as a viable therapeutic option for CVD, results from long‐term anti‐sense oligonucleotide (ASO) studies have raised questions about the possible deleterious metabolic consequences of antagonizing miR‐33 (Goedeke *et al,* 2013). Moreover, genetic knockout of miR‐33 was found to promote obesity and metabolic dysfunction, which was greatly exacerbated by HFD feeding (Horie *et al,* 2013). Further characterization of an independent miR‐33 knockout mouse model showed similar effects on body weight and glucose homeostasis along with impaired function in metabolic tissues like white adipose tissue and the liver. This work also demonstrated that the predisposition to obesity and metabolic dysfunction was due to an increase in food intake, as no differences in body weight or metabolic function were observed when miR‐33‐deficient animals were pair fed to control animals (Price *et al,* 2018). This indicates that loss of miR‐33 may impact the regulation of feeding behavior either through direct effects on the arcuate nucleus of the hypothalamus or through indirect effects on the peripheral tissues that send signals to this region of the brain. In future work, it will be critical to determine what cells/tissues primarily contribute to these effects and the specific mechanisms by which miR‐33 mediates these changes.

### Regulation of non‐cardiometabolic diseases by miR‐33

In addition to its important role in the regulation of atherosclerosis, obesity, and other metabolic conditions, further work on miR‐33 has demonstrated its ability to regulate a diverse array of physiologic functions related to different disease states, including immune response, cellular proliferation, and cognition. miR‐33 is highly expressed in the brain and plays an important role in regulating cognitive function both directly and indirectly. Through targeting of multiple factors involved in GABAergic signaling, miR‐33 has been shown to directly regulate state‐dependent memory (Jovasevic *et al,* 2015). Additionally, multiple groups have shown that miR‐33 can promote amyloid‐beta accumulation, indicating that inhibition of miR‐33 may protect against memory loss in patients with Alzheimer’s disease (Kim *et al,* 2015; Wang *et al,* 2020).

Recent work by a number of different groups has also demonstrated that miR‐33 plays an important role in regulating both innate and adaptive immunity, which can have an important impact on response to infectious diseases, as well as conditions associated with systemic inflammation, as discussed in relation to atherosclerosis. In macrophages, miR‐33 has been shown to be regulated in response to infectious stimuli (Zhao *et al,* 2014; Lai *et al,* 2016; Ouimet *et al,* 2016b; Liu *et al,* 2019), and inhibition of miR‐33 has been shown to provide protection against both bacterial and viral infections (Ouimet *et al,* 2016b; Liu *et al,* 2019). Additionally, miR‐33 has been shown to regulate adaptive immunity through impairment of T‐cell priming, leading to impaired functional capacity of memory T cells (Klein Geltink *et al,* 2017).

miR‐33 regulates cell cycle progression by targeting CDK6, CCND1, and p53, thereby arresting cells in G1 phase and inhibiting cell growth (Herrera‐Merchan *et al,* 2010; Cirera‐Salinas *et al,* 2012). This has been shown to impact multiple different cell types and processes including myeloid cell differentiation (Baba *et al,* 2018), adipocyte differentiation (Price *et al,* 2016), osteoblast differentiation (Wang *et al,* 2018), liver regeneration (Cirera‐Salinas *et al,* 2012), and tumor growth/metastasis (Zhang *et al,* 2015; Tian *et al,* 2016; Karatas *et al,* 2017; Huang *et al,* 2018; Weihua *et al,* 2020). SREBP expression is elevated in a number of malignancies and is a predictor of poor prognosis in patients (Cheng *et al,* 2018). Intriguingly, miR‐33a/b expression has been shown to be dissociated from their host genes in this context and act as tumor suppressors in lung cancer, breast cancer, pancreatic cancer, osteosarcoma, melanoma, and prostate cancer, through targeting oncogenic and EMT‐related proteins (Zhang *et al,* 2015; Tian *et al,* 2016; Karatas *et al,* 2017; Huang *et al,* 2018; Amaar & Reeves, 2019; Weihua *et al,* 2020; Xu *et al,* 2020).

Collectively, these studies demonstrate that in addition to cardiometabolic diseases, there are a wide range of other physiologic functions and disease states regulated by miR‐33. These findings have highlighted the likelihood that use of miR‐33 inhibitors to reduce the formation of atherosclerotic plaques could have a significant impact on many different tissues and cell types and could result in detrimental effects, such as the development of metabolic dysfunction and enhanced tumor growth. These important concerns have driven a new wave of research, in which researchers have sought to better understand the specific roles of miR‐33 in different tissues and the mechanisms by which miR‐33 mediates its effects. These recent studies into the specific functions of miR‐33 utilize a host of the new tools and strategies that have been developed to better understand miRNA biology as well as novel approaches to develop more targeted therapeutic strategies to minimize potential unintended effects.

## New approaches to better understand the specific roles of miR‐33

### Assessing cell and tissue‐specific roles of miR‐33 in the regulation of atherosclerosis

#### Macrophage miR‐33 in regulation of atherosclerosis

Initial efforts to assess the role of miR‐33 in specific tissues and subtypes were focused on determining the primary mechanisms by which miR‐33 regulates atherosclerosis. miR‐33 has been shown to regulate numerous functions related to plaque formation. By performing bone marrow (BM) transplants from miR‐33‐deficient animals into ApoE‐deficient mice (*Apoe* ^‐/‐^), Horie *et al* showed that both BM‐specific and whole‐body loss of miR‐33 resulted in reduced lipid accumulation in atherosclerotic plaques, but the effects on plaque size were more pronounced in animals lacking miR‐33 globally. These findings suggested that both liver and macrophages play an important role in mediating the effects of miR‐33 on atherosclerosis (Horie *et al,* 2012). However, the fact that both HDL‐C levels and RCT are dramatically reduced in *Apoe^‐/‐^* mice could have an important impact on these findings. More recent work using the *Ldlr*‐deficient mouse model of atherosclerosis did not observe any differences in atherosclerosis when miR‐33 was removed globally (Price *et al,* 2017). Reconstitution with miR‐33‐deficient BM was found to significantly reduce both lipid accumulation and plaque size, indicating that the beneficial effects of miR‐33 deficiency in macrophages were offset by the obesity and insulin resistance observed in these animals. This work demonstrated that loss of miR‐33 in macrophages promotes RCT *in vivo* leading to reduced lipid accumulation, as well as reduced inflammatory response and improved efferocytosis (Price *et al,* 2017).

#### Hepatic miR‐33 in atherosclerosis

These findings clearly demonstrate that loss of miR‐33 in macrophages is sufficient to reduce atherosclerotic plaque size, and suggest that enhanced macrophage cholesterol efflux may be the primary mechanism by which miR‐33 mediates these effects. However, the major metabolic alterations observed in animals lacking miR‐33 globally introduce numerous confounding factors that precluded any meaningful assessment of how regulation of HDL biogenesis and RCT in the liver may contribute to the effects of miR‐33 on atherosclerosis. Using a liver‐specific miR‐33 knockout mouse model, our recent work has demonstrated that hepatic loss of miR‐33 increases circulating HDL‐C and improves RCT in chow‐fed mice. Importantly, mice lacking miR‐33 only in the liver did not demonstrate the adverse metabolic effects observed in global miR‐33 knockout models. However, despite the beneficial changes in lipid metabolism, loss of miR‐33 in the liver did not have any impact on atherosclerotic plaque size (Price *et al,* 2021). This is likely due to the fact that SREBP2 and miR‐33 are strongly downregulated in the liver under hyperlipidemic conditions (Rayner *et al,* 2010; Nishino *et al,* 2018) and under these conditions liver‐specific miR‐33 knockout mice no longer show any differences in circulating HDL‐C or total cholesterol (Price *et al,* 2021). These findings support the conclusion that in rodents the pro‐atherogenic effects of miR‐33 are primarily due to direct effects on macrophages within the atherosclerotic plaque, but also indicate that detrimental effects of inhibition of miR‐33 in the liver are unlikely.

In humans, the impact of hepatic miR‐33 may be considerably more pronounced, as the second isoform of miR‐33, miR‐33b, is not found in rodents and may play a more prominent role in the liver under hyperlipidemic conditions. miR‐33b is encoded within the gene for SREBP1, which unlike SREBP2 is not repressed under hyperlipidemic conditions (Fig 1). Importantly, characterization of miR‐33b knock‐in mice demonstrated similar changes in miR‐33 isoforms, with miR‐33a being repressed and miR‐33b expression being increased in response to WD feeding (Nishino *et al,* 2018). Expression of miR‐33b in the liver was also found to be considerably higher, indicating that miR‐33b is the dominant miR‐33 isoform in the liver, especially under conditions likely to promote atherosclerosis. Consistent with this, mice expressing only miR‐33b (miR‐33b knock‐in in miR‐33a‐deficient animals) had significantly lower HDL‐C levels and larger atherosclerotic plaques than animals only expressing miR‐33a (Koyama *et al,* 2019). *Srebp1* is also known to be dysregulated under conditions of obesity and diabetes suggesting that miR‐33b expression may also be altered under these conditions.

### Dissecting the contribution of miR‐33 in different metabolic tissues in regulating glucose homeostasis and obesity

Considering the dramatic metabolic alterations in miR‐33‐deficient animals and the important implications of this for the potential use of miR‐33 inhibitors to treat atherosclerosis and other conditions, our recent work has sought to determine what organs and/or cell types are primarily responsible for driving these metabolic changes. Development of conditional miR‐33 knockout models has facilitated exploration of the specific roles of miR‐33 in key metabolic tissues (Price *et al,* 2021). As the work of Horie *et al* indicated that changes in hepatic lipid metabolism may be responsible for driving the metabolic dysfunction of miR‐33‐deficient animals (Horie *et al,* 2013), our initial efforts focused on characterizing the impact of hepatocyte‐specific miR‐33 deficiency, by crossing conditional miR‐33 knockout animals with mice expressing Albumin‐Cre. This work clearly demonstrates that loss of miR‐33 in the liver did not result in any differences in body weight, even after HFD feeding, and actually improved insulin sensitivity and regulation of glucose homeostasis. These animals also showed decreased expression with factors associated with inflammation and fibrosis after HFD feeding. These effects were likely mediated through derepression of both known targets of miR‐33 regulating fatty acid oxidation and novel targets involved in inhibition of fibrotic response (SKI). This protection from hepatic dysfunction was confirmed in a more acute model of hepatic damage (CCl_4_), providing further evidence that loss of miR‐33 in the liver can protect against the development of hepatic fibrosis (Price *et al,* 2021) (Fig 3).

Further attempts to determine what tissues and cell types may be responsible for driving the obesity and metabolic dysfunction associated with loss of miR‐33 have included the development of adipocyte (Adiponectin‐Cre)‐ and macrophage (LysM‐Cre)‐specific miR‐33 knockout models. Adipocyte‐specific deletion of miR‐33 did not result in any differences in body weight, regulation of glucose homeostasis, or response to a lipid challenge in HFD‐fed animals (Price *et al,* 2021). Bone marrow transplant from miR‐33‐deficient animals into LDLR knockout mice did not result in the obesity or metabolic dysfunction observed in miR‐33/LDLR double knockout animals, suggesting that macrophages and other hematopoietic cells were not responsible for driving this phenotype (Price *et al,* 2017). Consistent with this, characterization of macrophage‐specific miR‐33 conditional knockout mice also did not reveal any differences in body weight or circulating lipids under hyperlipidemic conditions (Price *et al,* 2021). In addition to demonstrating that neither direct effects of the liver, adipose tissue, or macrophages contribute substantially to the obesity phenotype of whole‐body miR‐33 knockout mice, this work also indicates that signaling from these peripheral tissues is not responsible for promoting the increased feeding that was observed. This suggests that either signals from other peripheral tissues or direct effects on the hypothalamic neurons that regulate feeding are likely responsible for the metabolic effects observed in miR‐33 knockout mice.

## Newly developed tools used to dissect the role of specific target mRNAs and therapeutic approaches to target miR‐33

### Identification and characterization of physiologically relevant miRNA targets

**Figure 4:**
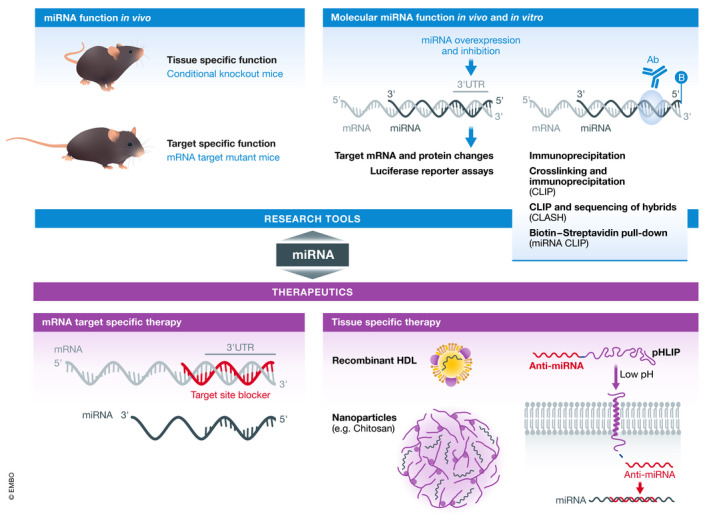
Novel research tools and therapeutic approaches to study miRNA biology Recent work has led to the development of new tools and techniques for studying miRNA targets and functions and therapeutically modulating miRNAs *in vivo*.

Recently, important advancements have been made in the development and implementation of new techniques for assessing physiologically relevant miRNA targets (Fig 4). The development of biotinylated miRNA constructs has facilitated the immunoprecipitation (IP) of specific miRNAs along with their target mRNAs (Orom & Lund, 2007). This approach was recently utilized to demonstrate that miR‐33 can directly regulate *Gabrb2* and *Kcc2,* important factors in GABAergic signaling that are involved in state‐dependent memory. While this technique provides a much more direct means of assessing miRNA binding to targets, it still requires transfection with artificial constructs, which limits the types of cell lines and conditions in which bindings can be assessed. On the other hand, recently developed techniques for IP of the RISC allow researchers to determine what mRNAs are associated with the RISC in physiologically relevant conditions and assess how the association of these mRNAs is impacted by alterations in the expression of specific miRNAs (Helwak & Tollervey, 2014). This is extremely important, as prior work has demonstrated that differences in the expression of both miRNAs and their targets can vary dramatically between tissues which can have a major impact on both the extent to which individual mRNAs are targeted and the impact of these effects. Additionally, other miRNAs can compete for targets in different tissues and molecules such as lncRNAs, which can act as sponges to bind miRNAs and impede their function. For example, recent work has identified the lncRNA, CHROME, as an important regulator of miR‐33 and other miRNAs involved in cholesterol metabolism (Hennessy *et al,* 2019).

The combination of these two techniques provides a much more direct and unbiased approach to identifying and assessing physiologically relevant miRNA targets than the methods previously employed. However, this strategy still does not directly assess the extent to which a specific miRNA target mediates the physiologic functions of a particular miRNA. This has been challenging because loss or overexpression of a miRNA impacts a number of different target mRNAs, and *in vitro* experiments often do not recapitulate what is observed *in vivo*. However, in our recent work we have described a new approach that utilizes new genome editing techniques to disrupt an individual miRNA/target interaction *in* vivo (Price *et al,* 2019b). As ABCA1 is a very strong target for miR‐33 and a critical factor in both cholesterol efflux and HDL biogenesis, we recently sought to determine the extent to which targeting of ABCA1 mediates the pro‐atherogenic effects of miR‐33. To accomplish this, we used CRISPR/Cas9 genome editing to selectively excise the region of the *ABCA1* 3’UTR containing three binding sites for miR‐33 and replace this with a construct in which the miR‐33 binding sites had been modified to prevent binding. Using this miR‐33/ABCA1 binding site mutant (BSM) mouse model, we were able to demonstrate that selective disruption of the ability of miR‐33 to target ABCA1 is sufficient to enhance macrophage reverse cholesterol transport and reduce atherosclerotic plaque burden *in vivo* (Price *et al,* 2019b). While it is still likely that the ability of miR‐33 to regulate factors involved in cellular bioenergetics and other functions related to atherosclerosis contributes to the beneficial effects of miR‐33 deficiency, these findings indicate that ABCA1 is a key driver that alone is capable of mediating the beneficial response. This was the first time that disruption of an individual miRNA/target interaction was demonstrated to have a significant impact on a complex physiologic response *in vivo*. Importantly, disruption of ABCA1 targeting by miR‐33 was not found to have any impact on body weight or metabolic function indicating that other miR‐33 targets are responsible for mediating these effects. This is important as it indicates that pharmacologic approaches to disrupt specific miRNA/target interactions could potentially provide a much safer strategy for modulating miRNA functions in human patients. Constructs designed to bind to specific miRNA binding sites in the 3’UTR of target genes are commercially available, but initial assessment has revealed issues with both functionality and specificity of these products. Therefore, the development of reliable and specific target site blockers would be a critical step forward for the miRNA field in terms of both research and the development of therapeutic strategies.

### Novel approaches to improve the targeting of miRNA therapeutics

Due to their promiscuous nature, miRNAs have been found to have a diverse range of effects in different cell type and tissues. As demonstrated in this review, this is especially true for miR‐33 and in some cases this may result in serious adverse outcomes. Because of this, a great deal of recent work in the miRNA field has been focused on the development of targeted delivery systems for miRNA therapeutics (Fig 4). One of the first examples of this for miR‐33 involved the use of an osteoblast‐specific liposome delivery system to specifically inhibit miR‐33 in these cells *in vivo* leading to protection against osteopenia (Wang *et al,* 2018). More recently, a number of different groups have demonstrated the potential for using targeted delivery systems to deliver miR‐33 inhibitors for the treatment of a number of different diseases.

Our prior work has shown that miR‐33‐deficient mice are protected against the development of kidney dysfunction (Price *et al,* 2019a). These effects were not due to changes in circulating leukocytes, as BM transplant from miR‐33 knockout mice was not found to be protective. More importantly, therapeutic inhibition of miR‐33, using pH low insertion peptides (pHLIPs) to deliver miR‐33 inhibitors specifically to the kidney and other acidic microenvironments, was also found to be protective against the development of kidney fibrosis (Price *et al,* 2019a). As other miRNAs have also been shown to play an important role in regulating the development of kidney fibrosis (Lv *et al,* 2018), the ability of pHLIPs to deliver miRNA mimics and inhibitors to the kidney could lead to the development of important therapeutic approaches for the development of chronic kidney dysfunction.

pHLIPs have previously been demonstrated to be an effective means of delivering miRNA inhibitors to the acidic microenvironment of tumors (Cheng *et al,* 2015; Sahraei *et al,* 2019), suggesting that these same constructs could be used for targeted delivery of miR‐33 therapeutics in this context. Considering the potential for disparate and possibly adverse effects in different organs, this type of targeted delivery system may prove incredibly valuable for miRNA‐based therapies. Additionally, lipid‐laden macrophages within atherosclerotic plaques have also been shown to have low pH, suggesting that these same delivery systems may also be useful for targeted delivery of inhibitors of miR‐33 and other miRNA therapeutics for the treatment of atherosclerosis. Indeed, our recent work has demonstrated that pHLIP‐conjugated inhibitors of miR‐33 can effectively target miR‐33 in the macrophages that make up atherosclerotic plaques leading to stabilization of the lesions (preprint: Zhang *et al,* 2020).

Recent research has also demonstrated that nanoparticle delivery systems can be used to promote more specific delivery of miRNA therapeutics. Chitosan nanoparticles were recently demonstrated to promote the uptake of miRNAs into macrophages both *in vitro* and *in vivo*, and delivery of miR‐33 using this system was found to impair macrophage‐mediated cholesterol efflux and RCT (Nguyen *et al,* 2019). Similarly, pH‐responsive and integrin‐targeting nanoparticles were shown to promote uptake of miR‐33 inhibitors into atherosclerotic plaques resulting in improved RCT and reduced plaque formation (Li *et al,* 2020).

As our work has demonstrated that loss of miR‐33 in the liver can protect against fibrosis and metabolic dysfunction, hepatocyte‐specific delivery of miR‐33 inhibitors may also have therapeutic value. Consistent with this, a recent study using mesoporous silica nanoparticles to deliver miR‐33 inhibitors showed a fivefold increase in liver uptake and a reduction in hepatic lipid accumulation (Tao *et al,* 2020). These findings are promising, and other approaches such as triantennary N‐acetylgalactosamine (GalNAc)‐conjugated anti‐sense oligonucleotides have also been demonstrated to be effective at targeted delivery specifically to the liver (Prakash *et al,* 2014).

## Conclusions and future directions

Since the discovery of miRNAs three decades ago, this field has advanced substantially, and yet we have only begun to scratch the surface of understanding how miRNAs regulate different physiologic functions and contribute to the development of different disease states. Additionally, more research is needed to understand how miRNA’s own regulation fits into the broader regulatory systems that control organismal and metabolic homeostasis. With the new tools at our disposal, researchers are positioned to begin addressing many of the most important questions hindering the field, which will be critical for continued progress in the development of clinical applications of miRNA research. In this review, we have sought to highlight both the major challenges facing the miRNA field and the approaches researchers are taking to overcome these issues using miR‐33, one of the most well‐studied miRNAs, as an example.

Recently, a great deal of progress has been made in understanding the specific role of miR‐33 in various tissues and determining the mechanisms by which miR‐33 regulates atherosclerosis and other conditions. These findings demonstrate the need for more advanced therapies designed to target specific areas in the body or only disrupt specific miRNA/target interactions, which could provide safer and more effective treatment options. Considerable progress has been made in the development of these types of techniques. Despite this, important questions remain about the mechanisms through which miR‐33 regulates obesity and metabolic function, and addressing these will be important for assessing the potential risks of miR‐33‐based therapeutic approaches in humans. In the future, more advanced delivery systems for miRNA therapeutics and further application of newly developed tools for improving our understanding of what mRNA targets are responsible for mediating the effects of miRNAs in different tissues and disease states will be essential for the continued progress of the challenging but incredibly important field of miRNA research.

## Conflict of interest

Carlos Fernández‐Hernando and Yajaira Suárez have a patent on the use of miR‐33 inhibitors to treat inflammation. The other authors report no conflicts.

**Pending issues**

- Determine the specific mechanisms by which loss of miR‐33 increases food consumption, leading to obesity and metabolic dysfunction.
- Validate the therapeutic potential of targeted miRNA therapies for the treatment of metabolic diseases.
- Develop improved pharmacologic approaches to disrupt specific miRNA/target interactions.
- Optimization of delivery approaches to overexpress and inhibit miRNAs in specific cells and tissues.

## For more information

https://www.heart.org: An international professional society that supports research on processes fundamental to cardiovascular disease (American Heart Association).

https://diabetes.org: An international professional society that supports research on processes fundamental to diabetes (American Diabetes Association).

https://omim.org/entry/612156: A resource page on miR‐33 from the Online Mendelian Inheritance in Man online catalog of human genes and genetic disorders.

http://www.mirbase.org: A searchable database of published miRNA sequences and annotation.

http://www.targetscan.org: A searchable database of predicted miRNA target genes.
